# The New Dental Law of Ohio

**Published:** 1893-07

**Authors:** E. J. Waye

**Affiliations:** Sandusky, O.


### The New Dental Law of Ohio.

BY E. J. WAYE, D.D.S., SANDUSKY, O.

In commenting upon the passage of this law the editor of a well-known dental journal said:—

"The Committee from the Ohio State Dental Society, having the measure in charge, and Senator McMakin, who introduced the bill, are deserving of great credit for the many excellent features it contains."

Possibly; and yet it is a little difficult to see what particular credit should attach to the presentation by the eminent Senator of a bill which, he was assured by this committee, would, if passed, work only good both for the dentist and the community.

It is doubtless a creditable thing to do right in all cases; but to do simply duty, under such circumstances, would seem hardly to merit extra enthusiasm.

To the committee, if *anybody*, would appear to be due whatever of praise should be bestowed, and whenever the results of its efforts have been carefully scanned whatever of credit is theirs should, and doubtless will be, freely bestowed.

The law for which the present one is a substitute reads as follows:

SECTION 4,404. "No person shall practice dentistry for compensation in Ohio without having received from the faculty of a dental college, of either this or a foreign country, a diploma or certificate of qualification issued by the State Dental Society.

"But when a person has been continuously engaged in the practice of dentistry for a period of five years previous to the passage of this law, he shall be considered to have complied with the provisions of this chapter."

Under the last provision of this law many men without a diploma, certificate or adequate qualifications became legalized practitioners, standing on the same legal plane with the graduate or the holder of a certificate from the State Board.

This was certainly mortifying, but the holders of these consoled themselves with the reflection that the "five-year-men"

once admitted, the door would be closed, and in future only the educationally qualified admitted to practice.

What was needed to make the old law perfectly satisfactory was the addition of a strict registration clause, under which all practitioners must present the evidence of their legal right to practice in Ohio, such evidence being the possession of a diploma from a reputable dental college, or a certificate from the State Board of Examiners. Such a registration act was secured, and met with general acceptance, but at the end was tacked on a provision by which "all persons who may have been regularly, since July 4, 1889, engaged in the practice of dentistry, of good moral character" (this last was well put, law breakers not being usually included in this class—and none but such desired the provision,) "shall be registered and licensed by said Board, as dentists, and shall receive a certificate of such registration and license, duly authenticated by the seal and signature of said Board."

Under the new law, the Board chosen from the State Dental Society was abolished, and a new one, with the same number of members, and having essentially the same powers, but which was appointed by the governor, took its place.

Whether the committee had lost confidence in the honesty and wisdom of the Ohio State Dental Society, whether it believed that the judgment of a single tariff man like the governor, who, whatever his ambitions, would hardly be likely to aspire to office in the Ohio State Society, and whose judgment, unbiased by such desire, might fairly be expected to be of an impartial character, while his long and brilliant career in political life so eminently fitted him to be a judge of men, and, of course of Dentists, who belong to that class, even although not all of them may be regarded as the best specimens of the genus homo. Whatever be the reason, the credit for the change in boards can in fairness only be ascribed to the committee in charge, and much good may it do them.

Leaving the committee with their "blushing honors thick upon them," let us turn to the board and ascertain, if possible, whether in their official capacity they have performed their duties.

After having, according to the statute, "registered, licensed, and presented with a duly authenticated certificate of registration, sealed and signed by the president and secretary of the board, "*all* persons who may have been *regularly*, since July 4, 1889, engaged (in defiance of law), in the practice of dentistry in this State, *of good moral character*," upon application and payment of a fee of two dollars, and upon producing satisfactory proof of the fact, "the Board is required to keep full minutes of all its proceedings, and a full register of all persons licensed and certified as dentists by said Board, which shall be public records and at all reasonable times open to inspection."

A transcript of any of the entries in such minutes and register, certified by the secretary under the seal of said Board shall at all times and places be competent evidence of the facts therein stated. "The certificate of the secretary of said Board of Dental Examiners, under the seal of said board, stating that any person is or is not a registered and licensed dentist shall be prima facie evidence that such person is, or is not, entitled to practice dentistry in this State."

Such being the law in the case, it would appear that as soon as possible after the period fixed as the limit for registration should have expired, a list of those registered should have been prepared and forwarded to every legalized practitioner, thereby putting him in possession of prima facie evidence upon which to base a complaint to the prosecuting attorney of his county.

More than a year has now elapsed since this law went into operation, and nearly that length of time has passed since the limit fixed for registration has expired.

Not only has no such list been made so far as known, but all inquiries concerning the proper course to pursue to secure the conviction of offenders, have been by the board disregarded. It may be because a stamp for return postage was not enclosed ; it may be that having complied with the provision by which the "full register of all persons licensed and certified as dentists" "shall at all reasonable times be open to inspection," its duties were considered as fully performed :—whatever the reason, the honorable and qualified dentists of Ohio, have for a whole year been deprived of information which was rightfully theirs, and thus prevented from taking measures for their protection under the law. Whether this was due to the neglect of the board, or the lack of forethought and wisdom on the part of the committee, is left for the consideration of those most interested.

---

The above communication, reflecting a little upon our recently enacted law, and upon those more immediately interested in securing its passage, and also upon the Board of Examiners, was written perhaps under a little misapprehension, or, at least, under a want of a full understanding of all the circumstances that surrounded this work. It may here be said that the bill, as finally passed by the legislature, was different in several particulars from the draught that was presented by the committee, and one which the committee with Senator McMakin and other members of the legislature sought to have enacted; but in the progress of the work it was found necessary to make some concessions or secure nothing; the former course was thought the better. The Board of Examiners which was appointed immediately after the enactment of the law proceeded promptly to the performance of the work devolving upon it under its requirements. The labor and difficulties attending this work were very great, and it was accomplished as promptly and, we think, discreetly and well as it could have been by any board.

A list of the registered dentists has been made, and will be in the hands of the profession of the State very soon. The Board was disposed to proceed without undue haste, in order that injustice should not be done to any. Some criticism has been made upon the board for issuing the certificate of the present board to those who held certificates from the board acting under the old law. These former certificates are, of course, valid and substantially recognized by this law, but it was supposed that many, if not all such persons being required to register would be pleased to have the certificate of the present Board, and the issuing of certificates to this class of persons was simply in obedience to such a supposed desire.

We think the law should be amended in two or three particulars; this, it is hoped, may be accomplished in the near future. The profession should rest content for the time being with the law in its present shape, and every one should use his influence to have its requirements obeyed. No dentist in the State should refuse to register who has proper attainments.—ED.

